# Letter to the editor from Thea and Buschard: “Progression from stage 1 to stage 3 type 1 diabetes characterized by hypoglycemia”

**DOI:** 10.1210/jcemcr/luag156

**Published:** 2026-07-02

**Authors:** Rikke Thea, Karsten Buschard

**Affiliations:** Faculty of Health and Medical Sciences, University of Copenhagen, Frederiksberg C DK-1870, Denmark; Faculty of Health and Medical Sciences, University of Copenhagen, Frederiksberg C DK-1870, Denmark

To the editor,

Why does hypoglycemia occur in a rapidly developing case of type 1 diabetes (T1D)? In their interesting article, Nagarapu and Felton present a case of a child with T1D who exhibited persistent hypoglycemia during stage 1 followed by rapid progression to stage 3 T1D [1]. The discussion underlines the current gap in knowledge regarding mechanisms driving hypoglycemia in early T1D and highlights α-cell dysfunction and dysregulated insulin release due to lymphocyte infiltration as potential contributors. However, the authors have no solid explanation for the case manifestations as we can present here.

The sphingolipid sulfatide is important for normal β-cell function and serves as a chaperone for insulin [2]. Sulfatide is abundant in healthy islets and is involved in insulin folding, granule stability, and regulated secretion. Notably, the C16 isoform of sulfatide has been shown to open KATP channels, which results in inhibition of insulin secretion [3]. For the individual β cell that has just secreted insulin, it is appropriate to have a period of relaxation to build up new insulin secretory granules close to the membrane. Lower levels of sulfatide function would therefore be expected to disinhibit insulin release and lead to periods of excessive insulin secretion.

As written in the article, autoantibodies serve as diagnostic tools but are not shown to have importance for the pathogenesis of T1D, but one autoantibody seems to be an exception. Antisulfatide antibodies are present in low amounts in healthy controls individuals but increase substantially at diabetes development [4]. Antisulfatide antibodies may block sulfatide and interfere with its function. Furthermore, high β-cell activity mediates relatively lower sulfatide levels [5]. At diagnosis, sulfatide content is immensely reduced in islets of T1D patients, showing values of only 23% of normal control levels [6]. This combination of decreased sulfatide availability and increased antibody-mediated interference may result in substantial loss of sulfatide-dependent modulation of insulin secretion. The resulting high β-cell activity may itself account for the rapid progression to the overt stage 3 T1D [7].

In the context of the case described by Nagarapu and Felton, such a mechanism could plausibly explain the presence of hypoglycemia despite the ongoing autoimmune process. If sulfatide is highly reduced in the early phase, the inhibition on the individual β cell on further insulin secretion due to sulfatide is not in play. The consequence is highly active β cells and thereby hypoglycemia due to massive insulin secretion. This explanation may be the case for the rapid transition from stage 1 to stage 3 in the mentioned patient [1].

## Data Availability

No new data were generated or analysed in support of this research.
